# A multicenter cohort study on mapping of lymph node metastasis for splenic flexural colon cancer

**DOI:** 10.1002/ags3.12620

**Published:** 2022-09-18

**Authors:** Jun Watanabe, Yukihide Kanemitsu, Hirokazu Suwa, Yoshihiro Kakeji, Soichiro Ishihara, Eiji Shinto, Heita Ozawa, Takeshi Suto, Junichiro Kawamura, Fumihiko Fujita, Michio Itabashi, Masayuki Ohue, Hideyuki Ike, Kenichi Sugihara, The Japanese Society for Cancer of the Colon and Rectum

**Affiliations:** ^1^ Department of Surgery, Gastroenterological Center Yokohama City University Medical Center Yokohama Japan; ^2^ Department of Colorectal Surgery National Cancer Center Hospital Tokyo Japan; ^3^ Department of Surgery Yokosuka Kyosai Hospital Yokosuka Japan; ^4^ Division of Gastrointestinal Surgery, Department of Surgery Kobe University Graduate School of Medicine Kobe Japan; ^5^ Department of Surgical Oncology, Graduate School of Medicine The University of Tokyo Tokyo Japan; ^6^ Department of Surgery National Defense Medical College Tokorozawa Japan; ^7^ Department of Colorectal Surgery Tochigi Cancer Center Utsunomiya Japan; ^8^ Department of Gastroenterological Surgery Yamagata Prefectual Central Hospital Yamagata Japan; ^9^ Department of Surgery Kindai University Faculty of Medicine Osakasayama Japan; ^10^ Department of Surgery Kurume University Hospital Kurume Japan; ^11^ Department of Surgery Institute of Gastroenterology, Tokyo Women's Medical University Tokyo Japan; ^12^ Department of Gastroenterological Surgery Osaka International Cancer Institute Osaka Japan; ^13^ Department of Surgery JCHO Yokohama Hodogaya Central Hospital Yokohama Japan; ^14^ Tokyo Medical and Dental University Tokyo Japan

**Keywords:** accessory middle colic artery, colorectal cancer, complete mesocolic excision, lymph node metastasis, splenic flexure

## Abstract

**Aim:**

There have been no reports of searching for metastases to lymph nodes along the accessory middle colic artery (aMCA). The aim of this study was to investigate the metastasis rate of the aMCA for splenic flexural colon cancer.

**Methods:**

Patients with histologically proven colon carcinoma located in the splenic flexure, clinically diagnosed as stage I‐III were eligible for this study. Patients were retrospectively and prospectively enrolled. The primary endpoint was frequency of lymph node metastasis to the aMCA (station 222‐acc and 223‐acc). The secondary endpoint was the frequency of lymph node metastasis to the middle colic artery (MCA) (station 222‐lt and 223) and left colic artery (LCA) (station 232 and 253).

**Results:**

Between January 2013 and February 2021, a total of 153 consecutive patients were enrolled. The location of the tumor was 58% in the transverse colon and 42% in the descending colon. Lymph node metastases were observed in 49 cases (32%). The presence of aMCA rate was 41.8% (64 cases). The metastasis rates of stations 221, 222‐lt, and 223 were 20.0%, 1.6%, and 0%, and stations 231, 232, and 253 were 21.4%, 1.0%, and 0%, respectively. The metastasis rates of stations 222‐acc and 223‐acc were 6.3% (95% confidence interval: 1.7%‐15.2%) and 3.7% (95% confidence interval: 0.1%‐19%), respectively.

**Conclusions:**

This study identified the distribution of lymph node metastases from splenic flexural colon cancer. If the aMCA is present, this vessel should be targeted for dissection, taking into account the frequency of lymph node metastasis.

## Graphical abstract

How often lymph node metastases occur in the lymph nodes along the accessory middle colic artery (aMCA) is unclear. This study showed that the metastasis rates of the intermediate lymph node (stations 222‐acc) and main lymph node (stations 223‐acc) of the aMCA were 6.3% and 3.7%, respectively, with the presence of aMCA (41.8%). If the aMCA is present, this vessel should be targeted for dissection, taking into account the frequency of lymph node metastasis.

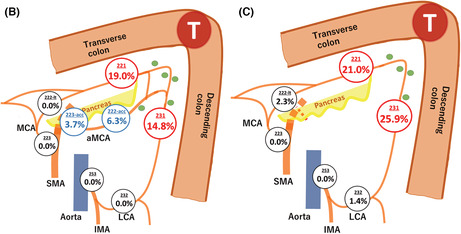

## 1 INTRODUCTION

Colorectal cancer is the second most common cancer worldwide and accounted for approximately 2.2 million new cases and 1.1 million deaths in 2019.^1^ Colon cancer of the splenic flexure was estimated to account for less than 10% of all colorectal cancers.^2^, ^3^, ^4^

The diversity of blood supply and lymphatic drainage of splenic flexure makes surgical management of splenic flexural colon cancer difficult, and the most appropriate surgical approach is still controversial.^5^ Colon cancer of the splenic flexure is thought to have several lymphatic drainage pathways. The lymphatic drainage pathway may be in the left branch of the middle colic artery (lt‐MCA) and left colic artery (LCA) regions.^6^ In addition, the accessary middle colic artery (aMCA) is an artery that branches off from the superior mesenteric artery (SMA), which is more proximal to the middle colic artery (MCA) and supplies the splenic flexure.^7^ The frequency of aMCA has been reported to be 4%‐49.2%,^7^ which is similar to that of the right colic artery.^8^

The Japanese Classification of Colorectal, Appendiceal, and Anal Carcinoma (JCCRC) defines lymph node groups and station numbers.^9^ The lymph node station in the large intestine is indicated with three‐digit numbers in the 200s. The station numbers associated with the splenic flexure are the pericolic lymph nodes of the MCA (station 221), intermediate lymph node of the lt‐MCA (station 222‐lt), main lymph nodes of the MCA (station 223), pericolic lymph nodes of LCA (station 231), intermediate lymph nodes of LCA (station 232), and main lymph nodes of the inferior mesenteric artery (IMA) (station 253). However, the JCCRC does not mention the aMCA and does not provide a lymph node station number.

Using indocyanine green (ICG) fluorescence imaging, Watanabe et al have demonstrated lymphatic flow along the aMCA in cases where the aMCA is present.^10^, ^11^ Their findings suggested that the lymphatic channels along the aMCA might be important pathways for the drainage of splenic flexural colon cancer, and dissection of the lymph nodes along the aMCA may result in improved oncological outcomes.^11^ However, there have been no reports investigating metastases to lymph nodes along the aMCA. How often lymph node metastases occur in the lymph nodes along the aMCA is thus unclear.

Therefore, the Japanese Society for Cancer of the Colon and Rectum (JSCCR), mainly composed of colorectal surgeons, conducted a multicenter cohort study to investigate the metastasis rate of the aMCA and to determine the optimal extent of lymph node dissection for splenic flexural colon cancer.

## 2 MATERIALS AND METHODS

### 2.1 Patients

This study was a multicenter, retrospective, prospective cohort study in Japan. The study protocol was approved by the Ethics Advisory Committee of Yokohama City University and the institutional review board of each participating hospital before the study was initiated and registered in the UMIN Clinical Trials Registry as UMIN‐CTR000037195 (http://www.umin.ac.jp/ctr/index.htm). Patients were recruited from 12 institutions of the JSCCR between January 2013 and February 2021. The patients encountered up to July 2019 were retrospectively accumulated, and those encountered from August 2019 were prospectively enrolled. For patients in the retrospective phase, we used the opt‐out approach to disclose the study information. For patients in the prospective phase, before enrolling in this study, all patients provided their written informed consent.

The eligibility criteria were as follows: (1) patients who were more than 20 years old; (2) patients with histologically proven colon carcinoma; (3) a tumor located within 10 cm from the splenic flexure; (4) a clinical diagnosis of Union for International Cancer Control (UICC) TMN classification (8th edition) stage I‐III.^12^; and (5) curative resection planned and scheduled for undergo lymph node dissection of ≥D2.

The exclusion criteria were as follows: (1) patients with distant metastasis (however, we did not exclude cases where distant lesions were incidentally identified during surgery); (2) patients with multiple cancer (except for Tis lesions); and (3) patients with a history of colorectal cancer resection.

### 2.2 Definition of lymph node station number

**FIGURE 1:**
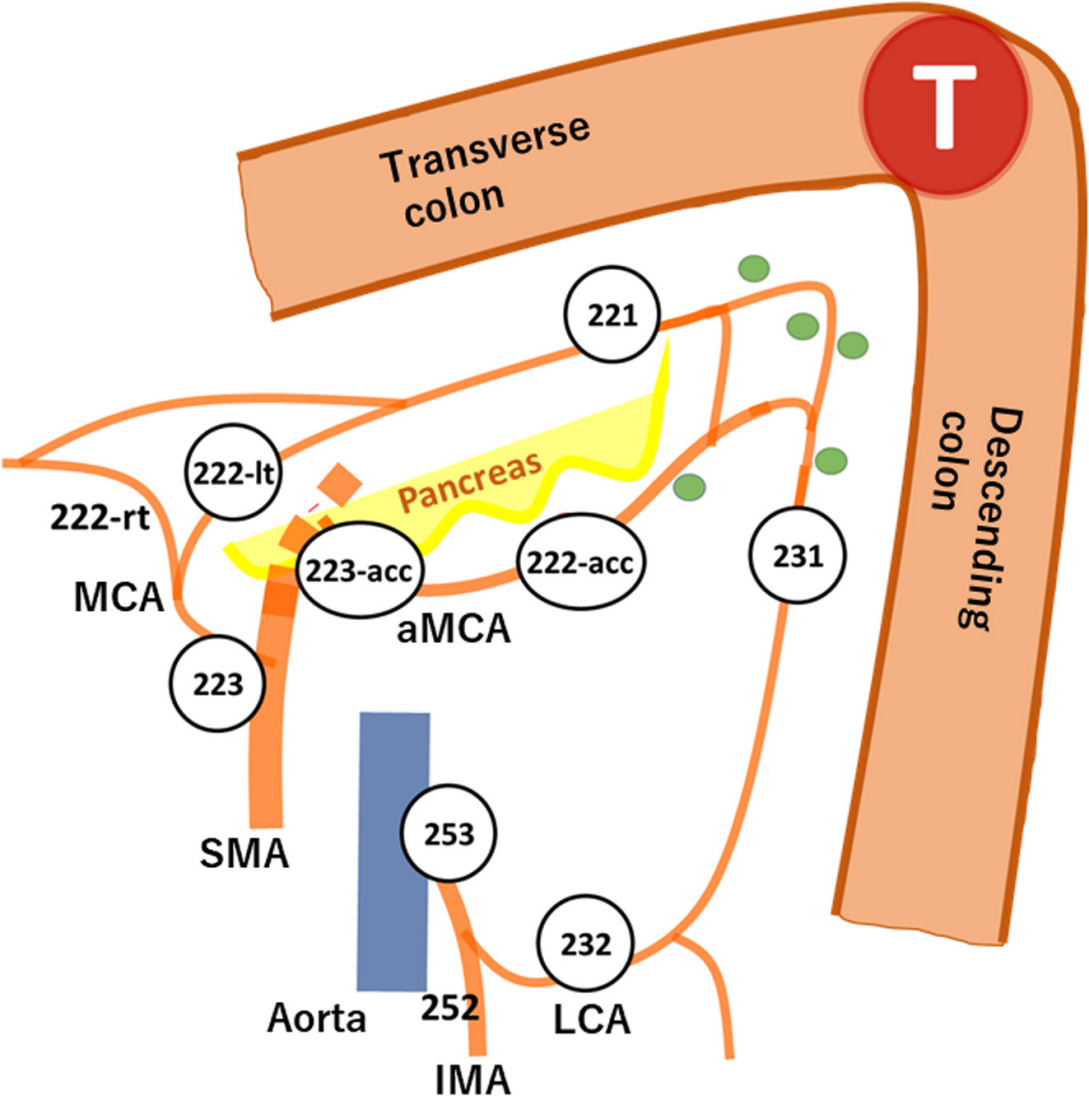
The definition of the lymph node station number. The lymph node station in the large intestine is indicated with three‐digit numbers in the 200s. the station numbers associated with the splenic flexure are pericolic lymph nodes of the MCA (station 221), intermediate lymph node of the lt‐MCA (station 222‐lt), main lymph nodes of the MCA (station 223), pericolic lymph nodes of LCA (station 231), intermediate lymph nodes of LCA (station 232), and main lymph nodes of the inferior mesenteric artery (IMA) (station 253). The intermediate lymph node of the aMCA was defined as station 222‐acc and the main lymph node of the aMCA was defined as station 223‐acc. aMCA, Accessory middle colic artery; IMA, Inferior mesenteric artery; LCA, Left colic artery; lt‐MCA, Left branch of middle colic artery; MCA, Middle colic artery; SMA, Superior mesenteric artery

**FIGURE 2:**
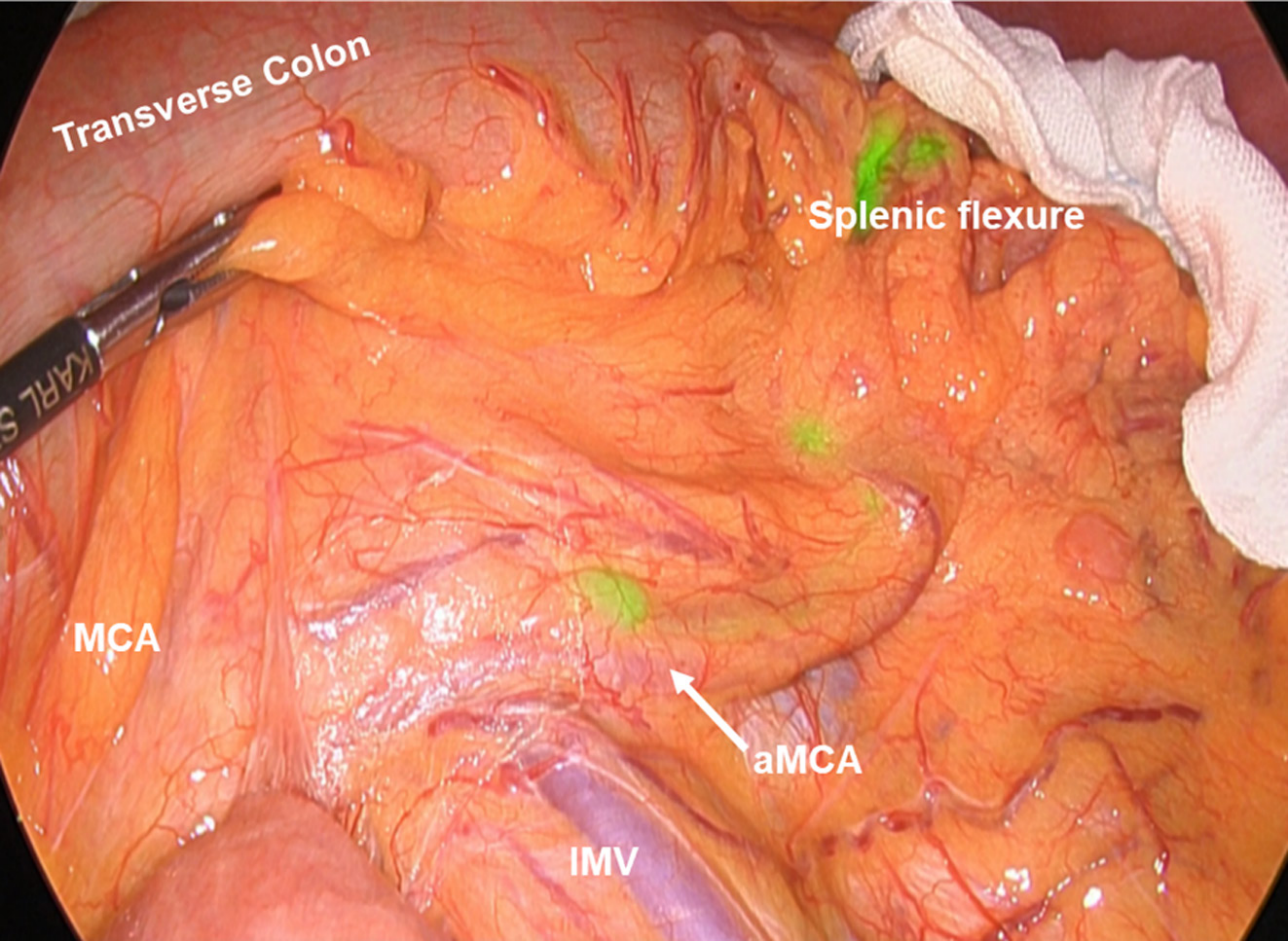
Definition of the accessory middle colic artery. The aMCA was defined as an artery running from the SMA to the splenic flexure branching from the SMA on the central side of the root of the MCA. Clinically, it was defined as an artery that crossed the anterior surface of the IMV and ran along the lower edge of the pancreas and toward the splenic flexure. aMCA, Accessory middle colic artery; SMA, Superior mesenteric artery; IMV, Inferior mesenteric vein

The definition of the lymph node station number is shown in Figure 1. Station numbers for the middle and left colic arterial regions followed the JCCRC.^9^ Since the station number of the aMCA was not defined in the JCCRC, the intermediate lymph node of the aMCA was defined as station 222‐acc, and the main lymph node was station 223‐acc. The aMCA was defined as the artery running from the SMA to the splenic flexure branching from the SMA on the central side of the root of the MCA. Clinically, it was defined as the artery that crossed the anterior surface of the inferior mesenteric vein and ran along the lower edge of the pancreas and toward the splenic flexure (Figure 2).

### 2.3 Procedure

For colon cancer located within 10 cm of the splenic flexure on preoperative imaging, segmental colectomy was performed according to the JCCRC.^9^ The large bowel dissection length was determined according to the following criteria: (A) when the feeding artery is directly below the tumor, the dissection area extends 10 cm in both the proximal and distal directions from the tumor margin; (B) when there is one feeding artery within 10 cm of the tumor margin, the dissection area extends up to 5 cm beyond the arterial inflow site, and the opposite side extends up to 10 cm from the tumor margin; (C) when there are two dominant arteries within 10 cm from the tumor margin, the dissection area extends up to 5 cm beyond the arterial inflow site in both the proximal and distal directions; (D) when there are no feeding arteries within 10 cm from the tumor margin, the dissection area extends up to 5 cm beyond the artery closest to the tumor margin, and the opposite side extends up to 10 cm from the tumor margin. Central vascular dissection was scheduled for ≥D2, as defined in the Japanese guidelines.^9^ Feeding arteries were identified by preoperative computed tomography and intraoperative findings. If the MCA was determined to be a feeding artery, the decision to dissect the lt‐MCA or the root of the MCA was left to the operator's discretion, depending on the clinical stage. At least station 222‐lt was dissected. Similarly, if the LCA was determined to be a feeding artery, the extent of LCA root dissection (whether or not to dissect up to station 253) was left to the operator's discretion, depending on the clinical stage. At least station 232 was dissected. If the aMCA was determined to be a feeding artery, the extent of the aMCA root dissection (whether the aMCA was dissected at the root or at the lower edge of the pancreas) was left to the operator's discretion, depending on the clinical stage. At least station 222‐acc was dissected. Whether the operation was open or laparoscopic had no influence on this decision.

### 2.4 Mapping of dissected lymph nodes

The surgeons identified lymph nodes from specimens after surgery and mapped them onto the schematic board, assigned numbers, and performed formalin fixation separately to compare the pathological findings based on the lymph nodal anatomical status.

### 2.5 Endpoints

The primary endpoint was the frequency of lymph node metastasis to the aMCA (station 222‐acc and 223‐acc). The secondary endpoint was the frequency of lymph node metastasis to the MCA (station 222‐lt and 223) and LCA (station 232 and 253).

### 2.6 Statistical analyses

The expected lymph node metastasis frequency to station 222‐acc of splenic flexural colon cancer was set to 5%, and the threshold lymph node metastasis frequency was set to 1%. A total of 139 patients would be required at a significance level of 0.05 and power of 0.80.

The data are presented as the median and interquartile range (IQR). We estimated metastasis rates and 95% confidence intervals (CIs) using the Clopper‐Pearson exact method. Statistical analyses were performed using the IBM SSPS Statistics software program, ver. 23.0 (IBM Corporation, Somers, NY, USA).

The full analysis set (FAS) was defined as all eligible patients. The metastasis frequency calculation was performed on the number of FAS population who underwent dissection of the station as the denominator and the number of FAS population who had pathological metastasis in the station as the numerator. The surgical and pathological outcomes was evaluated based on the FAS population.

## 3 RESULTS

A total of 153 consecutive patients from 12 hospitals were enrolled in this study. There were 88 patients with the retrospective accumulation and 65 with the prospective enrollment (clinicopathological characteristics and surgical outcomes regarding retrospective and prospective phase are presented in Tables S1 and S2). No patients were excluded, and the total 153 patients comprised the FAS population.

The clinicopathological characteristics of the patients are presented in Table 1. These patients included 67 females and had a median age of 70 years old and a median body mass index of 22.7 kg/m^2^. The performance status (Eastern Cooperative Oncology Group) in the majority of patients was grade 0. A total of 113 patients had comorbidities, and the American Society of Anesthesiologists (ASA) performance status in the majority of cases was ASA II. The location of the tumor was 58% in the transverse colon and 42% in the descending colon. Twenty‐six cases were located 5‐10 cm proximal, 62 cases 0‐5 cm proximal, 49 cases 0‐5 cm distal and 16 cases 5‐10 cm from the splenic flexure.

**TABLE 1:** Clinicopathological characteristics

		*n* = 153	
Age	Years	70	IQR: 60‐76.5
Sex	Male	86	56%
	Female	67	44%
BMI	kg/m^2^	22.7	IQR: 20.5‐24.4
ECOG‐PS	0	131	86%
	1	17	11%
	2	5	3%
ASA‐PS	I	32	21%
	II	110	72%
	III	11	7%
Tumor location 1	Transverse colon	88	58%
	Descending colon	65	42%
Tumor location 2	T: 5‐10 cm	26	17%
	T: 0‐5 cm	62	41%
	D: 0‐5 cm	49	32%
	D: 5‐10 cm	16	10%

Abbreviations: ASA‐PS, American Society of Anesthesiologists performance status; BMI, body mass index; D: 0‐5 cm, Tumor was located 0‐5 cm distal from the splenic flexure; D: 5‐10 cm, Tumor was located 5‐10 cm distal from the splenic flexure; ECOG‐PS, Eastern Cooperative Oncology Group performance status; IQR, interquartile range; T: 0‐5 cm, Tumor was located 0‐5 cm proximal from the splenic flexure; T: 5‐10 cm, Tumor was located 5‐10 cm proximal from the splenic flexure.

The surgical outcomes are presented in Table 2. The approach was open surgery in 25 cases and laparoscopic surgery in 125 cases. The degree of dissection was D2 in 28 cases and D3 in 125 cases. The median operative time was 221 min, and the median blood loss was 10 ml. The median tumor diameter was 40 mm. Proximal and distal margins were 93 and 105 mm, respectively. The median total number of retrieved lymph nodes was 19. Lymph node metastases were observed in 49 cases (32%), and lymph node metastases were pericolic lymph nodes in 42 cases, intermediate lymph nodes in six cases, and main lymph nodes in one case. The UICC TMN stage was stage I in 48 cases, stage II in 56 cases, and stage III in 49 cases. Postoperative complications (CD grade 2 or higher) were observed in 24 patients (16%), reoperation was observed in one patient (0.7%), and no mortalities were observed. The median length of postoperative hospital stay was 9 days.

**TABLE 2:** Surgical procedure and outcomes

		*n* = 153	
Approach	Open	25	16%
	Laparoscopic	128	84%
Dissection	D2	28	18%
	D3	125	82%
Operative time	Min	221	IQR: 177‐258
Blood loss	ml	10	IQR: 0‐36.5
Post‐op complication	≥CD grade2	24	16%
Re‐operation	(+)	1	0.7%
Mortality	(+)	0	0%
Post‐op hospital stay	Days	9	IQR: 7‐11
Tumor diameter	mm	40	IQR: 20.5‐50
Harvested LNs		19	IQR: 14‐26
Proximal margin	mm	93	IQR: 70‐115
Distal margin	mm	105	IQR: 80‐130
LNM	Total	49	32%
	Pericolic (n1)	42	27.5%
	intermediate (n2)	6	3.9%
	main (n3)	1	0.7%
UICC TNM Stage	I	48	31%
	II	56	37%
	III	49	32%

Abbreviations: IQR, interquartile range; LNs, lymph nodes; LNM, lymph node metastases; UICC, Union for International Cancer Control.

**FIGURE 3:**
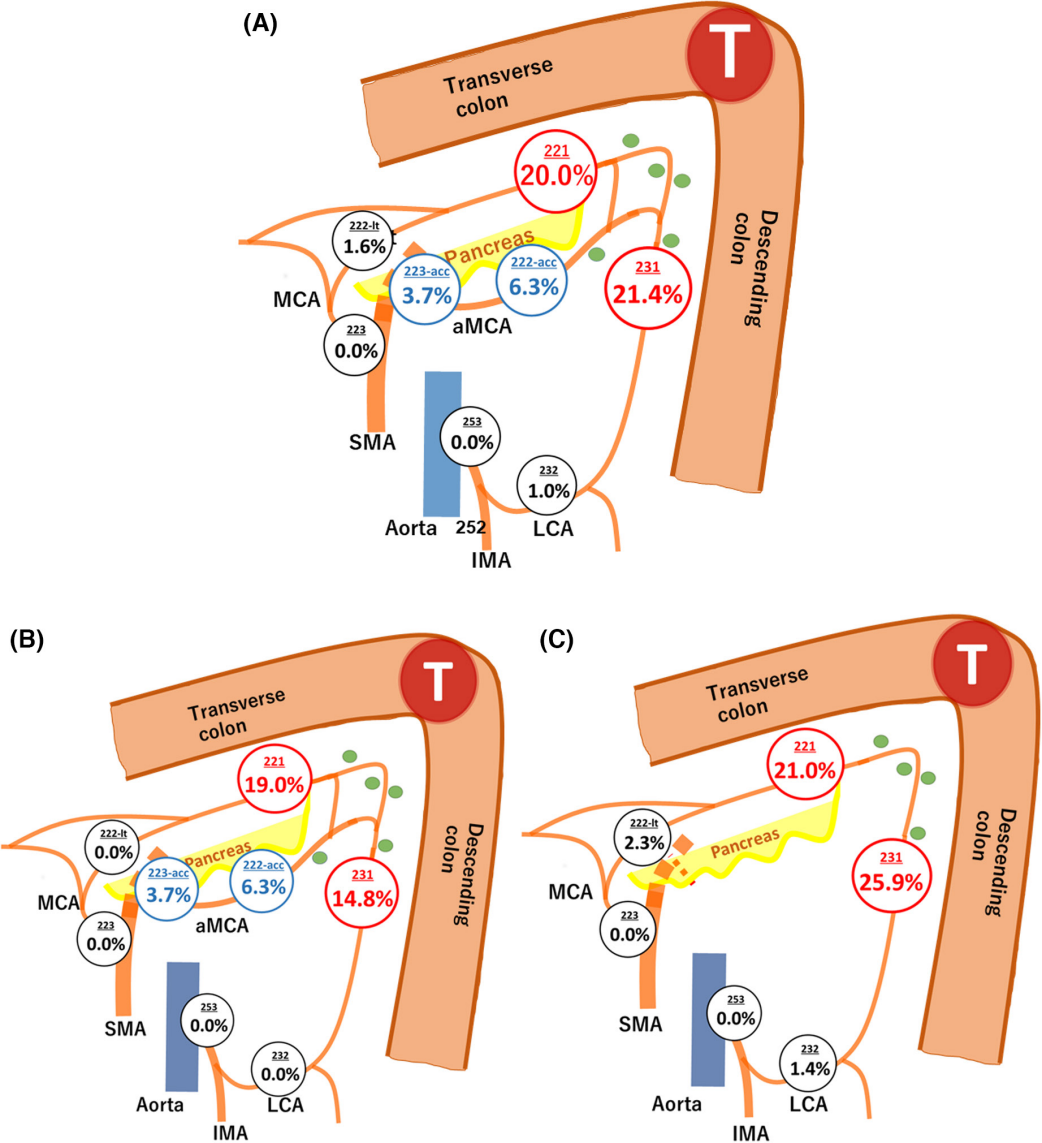
Metastasis rates of 153 patients who underwent surgical resection. (A) Overall population. (B) Metastasis rates of 64 patients with accessory middle colic artery. (C) Metastasis rates of 89 patients without accessory middle colic artery. aMCA, Accessory middle colic artery; IMA, Inferior mesenteric artery; LCA, Left colic artery; MCA, Middle colic artery; SMA, Superior mesenteric artery

We estimated the metastasis rates of targeted lymph nodes in the 153 patients who underwent curative resection (Table 3). The presence of aMCA rate was 41.8% (64 cases). The rate of lymph node metastasis was 30.0% (19/64) in patients with aMCA and 33.7% (30/89) in patients without aMCA. The metastasis rates of stations 221, 222‐lt, and 223 were 20.0%, 1.6%, and 0%, and those of stations 231, 232, and 253 were 21.4%, 1.0%, and 0%, respectively. The metastasis rates of stations 222‐acc and 223‐acc were 6.3% (95% CI: 1.7%‐15.2%) and 3.7% (95% CI: 0.1%‐19%), respectively (Figure 3A). No metastases were found at stations 222‐lt, 223, 232, or 253 in the presence of aMCA (Figure 3B,C). The lymph node metastasis rates in which the location of cancer was the transverse or descending colon are shown in Table 4.

**TABLE 3:** Metastasis rates of 153 patients who underwent surgical resection

	*N* = 153	Accessory middle colic artery	
Nodal station	Overall	Present	Not present
Overall	32.0% (49/153)	30.0% (19/64)	33.7% (30/89)
221	20.0% (24/120)	19.0% (11/58)	21.0% (13/62)
222‐lt	1.6% (1/61)	0.0% (0/17)	2.3% (1/44)
222‐acc	6.3% (4/64)	6.3% (4/64)	‐
223	0.0% (0/37)	0.0% (0/14)	0.0% (0/23)
223‐acc	3.7% (1/27)	3.7% (1/27)	‐
231	21.4% (28/131)	14.8% (8/54)	25.9% (20/77)
232	1.0% (1/104)	0.0% (0/33)	1.4% (1/71)
253	0.0% (0/61)	0.0% (0/16)	0.0% (0/45)

**TABLE 4:** Metastasis rates in which the location of cancer is the transverse colon or descending colon

	Transverse colon *N* = 88	Accessory middle colic artery	
Nodal station	Overall	Present	Not present
221	25.0% (22/88)	25.0% (11/44)	25.0% (11/44)
222‐lt	2.0% (1/50)	0.0% (0/15)	2.9% (1/35)
222‐acc	6.8% (3/44)	6.8% (3/44)	‐
223	0.0% (0/31)	0.0% (0/12)	0.0% (0/19)
223‐acc	5.3% (1/19)	5.3% (1/19)	‐
231	10.6% (7/66)	8.8% (3/34)	12.5% (4/32)
232	0.0% (0/48)	0.0% (0/19)	0.0% (0/29)
253	0.0% (0/19)	0.0% (0/8)	0.0% (0/11)

	Descending colon *N* = 65	Accessory middle colic artery	
Nodal station	Overall	Present	Not present
221	6.3% (2/32)	0.0% (0/14)	11.1% (2/18)
222‐lt	0.0% (0/11)	0.0% (0/2)	0.0% (0/9)
222‐acc	5.0% (1/20)	5.0% (1/20)	‐
223	0.0% (0/6)	0.0% (0/2)	0.0% (0/4)
223‐acc	0.0% (0/8)	0.0% (0/8)	‐
231	32.3% (21/65)	25.0% (5/20)	35.6% (16/45)
232	1.8% (1/56)	0.0% (0/14)	2.4% (1/42)
253	0.0% (0/42)	0.0% (0/8)	0.0% (0/34)

## 4 DISCUSSION

This is the first study conducted by the JSCCR to investigate the site and frequency of lymph node metastasis of splenic flexural colon cancer, taking into account the presence of the aMCA.

Blood supply to the splenic flexure has been shown to be variable. In 89% of cases, blood is supplied primarily by the IMA via the LCA, and in 11% of cases, it is supplied by the SMA via the MCA.^13^ The LCA and lt‐MCA are considered feeding arteries and the focus of lymph node dissection. This idea is also adopted in the Japanese guidelines, and the lymph nodes along the MCA are assigned station numbers in the 220s, while those along the LCA are assigned station numbers in the 230s.^9^ The aMCA was first reported by Steward et al in 1933.^14^ It has since been evaluated by dissecting cadavers^14^, ^15^, ^16^, ^17^, ^18^, ^19^ and based on intra‐operative findings.^11^ Although the detection rate of the aMCA was low before 1989 (8%‐21%), the rate has increased since 1990 (38.7%‐49.2%).^20^ In this study, the aMCA was present in 64 (41.8%) patients, which is consistent with the result of previous studies. In addition, in 2017, Watanabe et al reported their findings made by observing the lymphatic flow of the splenic flexure using ICG fluorescence imaging.^11^ In all cases with an aMCA, lymphatic flow along the aMCA was observed, suggesting that lymphatic channels along the vessel might be important pathways for drainage of splenic flexural colon cancer. Since there is no description of the aMCA in the Japanese Classification, lymph node mapping of the aMCA following surgery has not been performed. As a result, the frequency of lymph node metastasis along the aMCA has been unclear, even in Japan, where postoperative lymph node mapping is common.^21^, ^22^ In this study, in cases with the presence of the aMCA, 6.3% of intermediate lymph nodes (station 222‐acc) and 3.7% of main lymph nodes (station 223‐acc) had lymph node metastases. These data suggest that in cases where the aMCA is present, the aMCA should be targeted for central vascular ligation. In addition, in the presence of the aMCA, no metastasis of lt‐MCA and LCA to the intermediate lymph nodes was observed. In such cases, central vascular ligation of the lt‐MCA and LCA may not be needed. However, in the absence of the aMCA, the rate of metastasis to the intermediate lymph node of the lt‐MCA (station 222‐lt) is 2.3%, and the rate of metastasis to the intermediate lymph node of the LCA (station 232) is 1.4%, which is consistent with the results of previous studies.^21^, ^22^ If the aMCA is absent, the lt‐MCA and LCA should be considered for lymph node dissection.

According to a meta‐analysis of the surgical anatomy of the aMCA, the typical origin of the aMCA is the SMA on the central side of the MCA, but other less common sites of origin include the celiac trunk (3.6%), the dorsal pancreatic artery (2.1%), the common hepatic artery (1.9%), the jejunal artery (1.7%), the IMA (0.8%), the splenic artery (0.7%), right hepatic artery (0.7%), and the gastroduodenal artery (0.6%).^23^ In this study, we did not find any aMCAs exhibiting these aberrant branches. However, when ligating the aMCA at the root, we need to be aware that these aberrant branches may be present.

There are some limitations associated with this study. First, this study included patients collected in both a retrospective and a prospective phase. Second, this study was conducted to investigate the frequency of lymph node metastases and did not investigate long‐term outcomes. Third, the frequency of lymph node metastases along the aMCA that is given in this study has a wide CI because of the combination of a small number of patients with lymph node metastases and a small number of patients with aMCA.

In conclusion, this was the first study to investigate the frequency of lymph node metastases along the aMCA for splenic flexural colon cancer. If the aMCA is present, this vessel should be targeted for dissection, taking into account the frequency of lymph node metastasis.

## DISCLOSURES

Funding: None.

Conflicts of interest: Authors declare no conflict of interests for this article. The authors, Jun Watanabe and Yoshihiro Kakeji, are an editorial member of *Annals of Gastroenterological Surgery*.

## Supporting information


**Table S1** **and S2**
