# Supplementary material for: A multicenter cohort study on mapping of lymph node metastasis for splenic flexural colon cancer
Source: Ann Gastroenterol Surg. 2022 Sep 18;7(2):265–71. doi: 10.1002/ags3.12620 (PMC10043763; doi:10.1002/ags3.12620)
Supplement: Supplementary file 1 — Table S1 and S2 [file AGS3-7-265-s001.docx]

Supplementary Table 1. Clinicopathological characteristics regarding retrospective and prospective phase

|  |  | n=153 | | |
| --- | --- | --- | --- | --- |
|  |  | Retrospective phase  n=88 | Prospective phase  n=65 | |
| Age | Years | 70 | | 70 |
| Sex | Male | 50 (56.8%) | | 36 (55.4%) |
|  | Female | 38 (43.2%) | | 29 (44.6%) |
| BMI | kg/m^2^ | 22.5 | | 22.9 |
| ECOG-PS | 0 | 76 (86.4%) | | 55 (84.6%) |
|  | 1 | 8 (9.1%) | | 9 (13.8%) |
|  | 2 | 4 (4.5%) | | 1 (1.5%) |
| ASA-PS | I | 14 (15.9%) | | 18 (27.7%) |
|  | II | 69 (78.4%) | | 41 (63.1%) |
|  | III | 5 (5.7%) | | 6 (9.2%) |
| Tumor location 1 | Transverse colon | 55 (62.5%) | | 33 (50.8%) |
|  | Descending colon | 33 (37.5%) | | 32 (49.2%) |
| Tumor location 2 | T: 5-10 cm | 12 (13.6%) | | 14 (21.5%) |
|  | T: 0-5 cm | 42 (47.7%) | | 20 (30.8%) |
|  | D: 0-5 cm | 23 (26.1%) | | 26 (40%) |
|  | D: 5-10 cm | 11 (12.5%) | | 5 (7.7%) |

BMI: body mass index, ECOG-PS: Eastern Cooperative Oncology Group performance status, ASA-PS: American Society of Anesthesiologists performance status: T: 5-10 cm: Tumor was located 5-10 cm proximal from the splenic flexure, T: 0-5 cm: Tumor was located 0-5 cm proximal from the splenic flexure, D: 0-5 cm: Tumor was located 0-5 cm distal from the splenic flexure, D: 5-10 cm: Tumor was located 5-10 cm distal from the splenic flexure.

Supplementary Table 2. Surgical procedure and outcomes regarding retrospective and prospective phase

|  |  | n=153 | |
| --- | --- | --- | --- |
|  |  | Retrospective phase  n=88 | Prospective phase  n=65 |
| Approach | Open | 13 (14.8%) | 12 (18.5%) |
|  | Laparoscopic | 75 (85.2%) | 53 (81.5%) |
| Dissection | D2 | 13 (14.8%) | 15 (23.1%) |
|  | D3 | 75 (85.2%) | 50 (76.9%) |
| Operative Time | Min | 202.5 | 240 |
| Blood loss | ml | 4.5 | 14 |
| Post-op complication | ≥ CD grade2 | 13 (14.8%) | 11 (17.0%) |
| Re-operation | (+) | 0 (0.0%) | 1 (1.5%) |
| Mortality | (+) | 0 (0.0%) | 0 (0.0%) |
| Post-op hospital stay | days | 9 | 9 |
| Tumor diameter | mm | 35 | 40 |
| Harvested LNs |  | 18.5 | 21 |
| Proximal margin | mm | 92.5 | 97.0 |
| Distal margin | mm | 105 | 105 |
| LNM | Total | 31 (35.2%) | 18 (27.7%) |
| UICC TNM Stage | I | 28 (31.8%) | 20 (30.7%) |
|  | II | 33 (37.5%) | 23 (35.3%) |
|  | III | 27 (30.7%) | 22 (33.8%) |
| Accessory middle colic artery | present | 35 (40.0%) | 29 (44.6%) |

LNs: lymph nodes, LNM: lymph node metastases, UICC: Union for International Cancer Control
